# Cross-cultural comparison of causal attributions and help-seeking recommendations for mental illness : A Multinational Population-Based Study from 16 Arab Countries and 10,036 Individuals

**DOI:** 10.1192/j.eurpsy.2023.746

**Published:** 2023-07-19

**Authors:** M. Stambouli, F. Fekih Romdhane, A. Jaoua, Y. Boukadida, S. Ellini, M. Cheour

**Affiliations:** Psychiatry department E, Razi hospital, Manouba, Tunisia

## Abstract

**Introduction:**

Causal attributions of mental illness and help-seeking recommendations have multiple attitudinal and behavioral consequences; however, these factors have been subject of limited research in our Arab Muslim context.

**Objectives:**

This study examined causal attributions and help-seeking recommendations for mental illnesses among a large sample of the general population in Arab countries.

**Methods:**

We carried out a multinational cross-sectional study using online self-administered surveys in the Arabic language from June to November 2021 across 16 Arab countries.The Community Attitudes toward the Mentally Ill scale,the Mental Health Knowledge Schedule scale and the Attitudes Toward Seeking Professional Psychological Help Scale-Short Form were administered to participants from the general public.

**Results:**

The study sample was predominantly female (77%), married (41%), educated (89% with tertiary education), living in urban areas (85%), with a mean age of 29.6 ± 10.8 years.

Psychosocial causes including lack of parental affection (88.0%) and childhood sexual abuse (85.5%) were the most common causal attributions of mental illnesses endorsed by our participants, with 95.7% of them agreeing with at least any one of the psychosocial causes.

Palestinians were the most inclined to believe that mental illness is caused by Jinn possession and Magic/witchcraft (65.9% and 68.1%, respectively), followed by Algerians (56.2% and 68.6%, respectively), Kuwaitis (52.3%, and 62.7%, respectively), Yemenis (50.2%, and 61.4%, respectively) and Saudi participants (49.7%, and 61.2% respectively); whereas Tunisians were the least inclined to believe in these causes (18.6%, and 21.6%, respectively) (Table S3, supplemental material).

Even though most of the study subjects tended to have a higher preference to seek help from formal sources than informal sources, they showed a high propensity to some informal sources such as family members (80.4%) and confidants (68.6%). Besides, Algerians were the most likely to ask help from a cleric or traditional healers (68.6% and 69.9%), followed by Palestinians (61.8% and 65.3%, respectively), Egyptians (58.4% and 48.8%), Jordanians (57.7% and 64.2%) and Kuwaitis (57.0% and 61.9%).

**Conclusions:**

Interventions aiming at improving help-seeking attitudes and behaviors and promoting early access to care are required to be culturally tailored, and congruent with public beliefs about mental illnesses and their causations.

**Disclosure of Interest:**

None Declared

